# *Pantoea* spp. Associated with Smooth Crabgrass (*Digitaria ischaemum*) Seed Inhibit Competitor Plant Species

**DOI:** 10.3390/microorganisms7050143

**Published:** 2019-05-21

**Authors:** Matthew T. Elmore, James F. White, Kathryn L. Kingsley, Katherine H. Diehl, Satish K. Verma

**Affiliations:** 1 Department of Plant Biology, Rutgers University, 59 Dudley Rd., New Brunswick, NJ 08901, USA; jwhite3728@gmail.com (J.F.W.); kathryn.l.kingsley@gmail.com (K.L.K.); katherine.diehl@rutgers.edu (K.H.D.); 2 Centre of Advanced Study in Botany, Banaras Hindu University, Varanasi 211005, India; skvermabhu@gmail.com

**Keywords:** biological control, bioherbicide, *Curvularia*, *Pantoea*, Pantoea ananatis, weed ecology

## Abstract

*Digitaria ischaemum* (Schreb.) Schreb. ex Muhl. and *Poa annua* L. are competitive, early successional species which are usually considered weeds in agricultural and turfgrass systems. Bacteria and fungi associated with *D. ischaemum* and *P. annua* seed may contribute to their competitiveness by antagonizing competitor forbs, and were studied in axenic culture. *Pantoea* spp. were the most common bacterial isolate of *D. ischaemum* seed, while *Epicoccum* and *Curvularia* spp. were common fungal isolates. A variety of species were collected from non-surface sterilized *P. annua*. Certain *Pantoea* spp. isolates were antagonistic to competitor forbs *Taraxacum officinale,* *Trifolium repens*. All bacterial isolates that affected *T. officinale* mortality were isolated from *D. ischaemum* seed while none of the *P. annua* isolates affected mortality. Two selected bacterial isolates identified as *Pantoea ananatis* were evaluated further on *D. ischaemum,* *T. repens* (a competitor forb) and *P. annua* (a competitor grass) alone and in combination with a *Curvularia* sp. fungus. These bacteria alone caused >65% *T. repens* seedling mortality but did not affect *P. annua* seedling mortality. These experiments demonstrate that *Pantoea* *ananatis* associated with *D. ischaemum* seeds is antagonistic to competitor forbs in axenic culture. The weedy character of *D. ischaemum* could at least in part stem from the possession of bacteria that are antagonistic to competitor species.

## 1. Introduction

*Digitaria* (crabgrass) species are some of the most competitive C_4_ weeds of agricultural, horticultural and turfgrass landscapes in tropical and temperate regions. *Digitaria ischaemum* and *D. sanguinalis* (smooth and large crabgrass, respectively) are among most problematic weed species in the United States, and are particularly well adapted to turfgrass systems where as a C_4_ species they often outcompete C_3_ grasses and forbs in the summertime [1]. *Digitaria sanguinalis* is known to produce allelochemicals that may contribute to its competitiveness, but these have not been studied extensively [2,3].

*Poa annua* is another prolific early successional C_3_ plant that is extremely competitive in turfgrass and horticultural systems and can be found on all seven continents [4]. The success of *P. annua* as a weed is attributed to genotypic variability, prolific seed production, and a short life cycle [5].

The weed microbiome may also contribute to weed competitiveness. Communities of bacteria and fungi which colonize internal tissues as commensals or mutualists are referred to as endophytes and are ubiquitous in plants (reviewed in [6,7,8]). A wide range of endophytes are commonly found in plants, although there are several potential mechanisms that reduce microbial endophyte density and diversity when compared with the root rhizosphere [6,9]. These endophytes can affect tolerance to abiotic and biotic stressors such as disease, drought, heat and salinity [10,11].

The well-studied *Epichloë* endophyte symbionts are known to confer enhanced drought tolerance and resistance to insect herbivory in grasses [12,13]. These fungi are vertically transmitted through seed of the host plant. It is thought that early-successional weeds may rely on associations with non-clavicipitaceous endophytes to increase their competitiveness, although these have not been studied extensively [14]. Two exceptions are endophytes associated with *Centaurea stoebe* and *Phragmites australis*, which can be invasive outside of their native range. A sampling of fungal endophytes associated with *C. stoebe* achenes (seeds) in the invaded range (North America) found that they were more diverse and were taxonomically different than those associated with plants in the native (European) range [15]. Other researchers found *C. stoebe* infected with a fungal endophyte from the native range and another endophyte common to the non-native range suppressed competitor grasses of the invaded range more than grasses in the native range [16]. There is considerable diversity among fungal endophytes of *P. australis* in North America as well, although the contribution of these endophytes to fitness of mature plants is unknown [17]. However, other researchers have demonstrated that *P. australis* seed-associated bacteria and fungi from native and invaded ranges can enhance germination, seedling growth and antagonize other species [18,19,20]. 

Although *Digitaria ischaemum and Poa annua* are not classified as invasive weeds like *P. australis* and *C. stoebe,* they are very prolific in North America outside of their native Eurasian range [1,21]. It is not known whether endophytes affect competitiveness of *Digitaria* spp. or *P. annua*. Endophytes of *P. annua* have not been reported, but this weed has been found to cause changes in the soil microbial community that affect the fitness of mid-successional species [22]. Previous research has examined fungal endophytes of *Digitaria* spp., but not seed-associated bacteria, and. found over 20 different fungal taxa endophytic to roots of *D. ischaemum* grown in a geothermal soil in China [23]. They found most of these endophytic fungi of *D. ischaemum* were also found in the rhizospheric soil, but there were some species found only in the plant, including *Curvularia protuberata*, suggesting endophytes were vertically or horizontally transmitted. Other researchers have demonstrated that *C. protuberata* colonized by a virus increases thermotolerance of *Dichanthelium lanuginosum* (panic grass) [24,25,26]. This previous research with *Digitaria* spp. endophytes did not examine their role in antagonizing competitor plants.

Compared to endophytes of root and shoot tissues, considerably less research has been conducted on seed-associated bacteria. Prolific seed production is a key strategy for *P. annua* and *D. ischaemum* survival. Where *P. annua* populations are high, seedbank densities have been reported at between 20,000 and 200,000 seeds per m^2^ [27]. *D. ischaemum* and *D. sanguinalis* are annuals that survive by prolific seed production and can produce up to 188,000 seeds per plant [1]. Bacterial endophytes have been isolated from seed of several food crop species including rice (*Oryza sativa*), maize (*Zea mays*) as well as certain grass species [7,28,29]. These endophytes can be important for seedling growth and development and can contribute to the endophytic community of a mature plant [30,31]. In forbs, fungal endophytes can be vertically transmitted from seed to true leaves in aseptic conditions after surface-sterilization [32]. In grasses, bacterial endophytes can be transmitted from mature maize parents to seed offspring [33]. It is possible that these endophytes are vertically transmitted because they benefit the host plant. Cultivable diazotrophic endophytes of *Pennisetum purpureum*, a perennial C_4_ plant that is capable of producing large amounts of biomass under low or high N_2_ fertilization were found to produce indole acetic acid and solubilize phosphate and may play a role in the competitiveness of this C_4_ grass in the wild [34]. 

We hypothesized that bacteria and fungi associated with *D. ischaemum* and *P. annua* seed antagonize competitor species and enhance the ability of host seedlings to grow. Therefore, the objectives of our research were to: (1) Identify seed-vectored bacteria and fungi, and (2) Determine the effect of these bacteria and fungi on competitor forbs as well as host species.

## 2. Materials and Methods

### 2.1. Seed Collection

*Digitaria ischaemum* seed was collected from a single site at the Rutgers Horticultural Research Farm No. 2 in North Brunswick, NJ (40°45′25″ N 74°47′67″ W). The site consisted of a mixed stand of *D. ischaemum* and *Lolium perenne* maintained as a mowed stand of turfgrass for several years. Mowing was suspended in October to allow inflorescence production and maturity prior to harvest in November 2016. Seed was collected from a 300 m^2^ area using a leaf sweeper. Seeds were stored at 13 °C until January 2017 where they were moved to storage at −20 °C for one week prior to preparation to break seed dormancy. *Poa annua* seed was collected in June 2016 from a single site in University Park, PA (40°81′09″ N 77°86′73″ W) that was maintained as a mowed turfgrass stand for several years. Seeds were stored at 13 °C until January 2017 where they were moved to storage at −20 °C for one week prior to preparation to break seed dormancy.

### 2.2. Surface Sterilization and Microbe Isolation

*P. annua* and *D. ischaemum* seeds were surface sterilized by placing 5 g of seed in a 200 mL container filled with a 160 mL solution of DI water and 4.125% (*v*/*v*) NaOCl based on the methods of [20]. This container was placed on an orbital shaker to vigorously agitate the solution for 40 min. The bleach solution was then decanted and seeds were rinsed at least five times with sterile DI water in a laminar flow hood. Seeds were then placed on Petri dishes containing yeast extract sucrose agar (YESA; 1% yeast extract, 1% sucrose, 1.2% agar) and the plates were incubated at room temperature. Four seeds were placed on each plate and there were 10 replicates. The process was repeated with *D. ischaemum* seed one week later with 8 replicates; a second 5 g sample of seed was sterilized using the same process except that 5 μL of polysorbate 20 (Tween20, Thermo Fisher Scientific, Waltham, MA, USA) was added to the 160 mL of 4.125% (*v*/*v*) NaOCl solution. Outgrowing fungi were observed after 48 h of incubation and isolated to pure culture on potato dextrose agar for further study. After 7 to 14 days of incubation, bacteria were observed and isolated to pure culture on Luria-Bertani (LB) agar for further study. No outgrowing fungi or bacteria were observed in surface-sterilized *P. annua* seed after 14 days of observation. Therefore, using the same methods described above except without NaOCl, *P. annua* seeds were agitated in sterile water for 5 min and then the water was decanted and seeds were rinsed with sterile water before placing on YESA to isolate bacteria from non-surface-sterilized seeds.

### 2.3. Bacterial Identification and Characterization

Genomic DNA from bacteria was isolated using GenElute Bacterial Genomic DNA Kits (SigmaAldrich, St. Louis, MO, USA). Bacterial identifications were made by obtaining 16S rDNAsequences after methods employed by [35] using universal primers 16SF (5′-AGAGTTTGATCCTGGCTCAG-3′) and 16SR (5′-CTACGGCTACCTTGTTACGA-3′). Amplified PCR products were resolved by electrophoresis in 1.5% (*w*/*v*) agarose gel stained with SYBR safe for visual examination. The PCR products were purified using a PCR purification kit (Qiagen, Germantown, MD, USA) and sent to Genewiz Inc. (South Plainfield, NJ, USA) for sequencing. For certain isolates determined to be of interest after initial experiments (*Pantoea* spp. isolates 4 and 5), the tuf gene was also sequenced. Amplification of rDNA was performed in a 25 µL volume comprising: 12.5 µL Taq NEB 2x Master Mix, 9.5 µL water, 1.0 µL forward tufGPR (5′-GATACCAGTTACGTCAGTTGTACGGA-3′), 1.0 µL reverse tufGPF (5′-ACGTTGACTGCCCAGGACAC-3′) and 1.0 µL DNA. The reaction was performed in a thermocycler with an initial denaturation at 95 °C for 3 min, followed by 35 cycles at 95 °C for 30 s, 55 °C for 1 min, 72 °C for 30 s, and a final extension at 72 °C for 5 min. Sequences were compared to GenBank accessions using BLASTn (Available online: http://www.ncbi.nlm.nih.gov). Sequences were deposited in GenBank under accession numbers MG100861 to 863 and MK733357 to 370.

### 2.4. Experiment 1: Effect of Bacterial Isolates on Taraxacum Officinale Seedling Mortality

In turfgrass systems, *Taraxacum officinale* is a common competitor forb and was selected for use in these experiments. *T. officinale* seeds (collected from a lawn in South River, NJ, USA) were surface sterilized using the same process described above for *D. ischaemum* surface sterilization (agitation with 4.125% NaOCl for 40 min). Ten T. officinale seeds were plated onto each experimental unit, which consisted of a Petri dish (85 mm diameter) filled with 0.7% agarose media with no mineral nutrients. Petri dishes were maintained at ambient room temperature (22 °C to 27 °C) 40 cm below a fluorescent light source emitting 65 µmol m^−2^ s^−1^ PAR for 16 h per day. Isolates were maintained on trypticase soy agar (TSA) and streaked onto LB agar 12 to 24 h before inoculation. The bacteria were then removed from the agar with an inoculation loop and suspended in 1 mL of sterile water. Using this method, the suspension typically measured 0.8 OD ± 0.1 (600 nm) when diluted 1:3 with sterile water. One 3 µL drop of bacterial suspension was pipetted onto each seed within 1 h of placement on agarose. Isolates listed in Table 1 was evaluated in triplicate on a total of 30 seeds. A non-treated axenic control was included for comparison. A *Pseudomonas fluorescens* (Sandy LB4; GenBank No. KX665565) and *Pantoea* sp. isolate (RiLB4; GenBank No. KX752781) isolated from *Phragmites australis* seed by [20] were included as standards of comparison. The Sandy LB4 isolate has been shown to increase mortality of competitor forbs including *T. officinale* while RiLB4 did not affect mortality.

*T. officinale* seedlings that germinated (radicle at least 1 mm in length) were assessed as healthy, injured or dead at 14 days after treatment. Seedlings were considered healthy if cotyledons and leaves were green in color, turgid and not displaying any symptoms of cell membrane leakage (necrosis or greasy, off-color leaf tissues). Seedlings were considered dead if they were completely necrotic. Seedlings were scored as injured if they displayed some injury symptoms (i.e., chlorosis, necrosis) but other sections of the seedling were healthy and the seedling was not completely dead. The number of healthy, injured, and dead seedlings was assessed in each Petri dish and this number was used to determine the percentage of healthy, injured or dead plants in each experimental unit. Isolates were inoculated on different dates. Therefore, separate non-treated axenic controls were used for each experimental run and comparisons were made only to the non-treated control within each experiment. For isolates that increased mortality compared to the non-treated control, this experiment was repeated on *T. officinale* and *T. repens*. *T. repens* is also a common competitor species in turfgrass. *T. repens* seeds (purchased from a commercial seed source) were surface-sterilized using a similar method as *T. officinale* except that a 2% (*v*/*v*) NaOCl solution was used instead of a 4.125% solution. To ensure this method effectively sterilized clover seed, 100 seeds were placed on LB agar and no bacteria or fungi were observed after 10 d of incubation. 

After *T. officinale* plants were evaluated at 14 days after treatments, the agarose plates containing seedlings were flooded with a 2 mmol/L solution of 3,3-diaminobenzidine tetrahydrochloride dissolved in DI water (DAB; Sigma Aldrich, St. Louis, MO) 12 h prior to observation to aid in visual observation of bacteria [36]. Randomly selected inoculated and non-inoculated plants were removed from the agarose and squash prepared with aniline blue (0.01%) dissolved in water and lactic acid for microscope observation at 1000× magnification. 

### 2.5. Experiment 2: Effect of Bacterial Isolates on D. ischaemum Germination and Seedling Mortality

Based on the results of Experiment 1, *Pantoea* spp. bacterial isolates (4, 5, 8, 18, and 22) were evaluated against peeled, surface-sterilized *D. ischaemum* seedlings. To successfully remove cultivable bacteria from *D. ischaemum* seeds required several iterations of various sterilization procedures before an effective method was found. Seeds were placed on mesh screen and a wooden block was used to rub the seeds through the plastic screen, which removed the paleas and lemmas. These seeds were then placed on a finer plastic screen and a wooden block wrapped in the same screen was rubbed on the seed to remove the seed coat. These peeled seeds were separated from the debris and put into a 2 mL microcentrifuge tube filled with 1.5 mL of a 1.5% (*v*/*v*) NaOCl solution. The tube was vortexed for 5 min, the NaOCl solution was removed and fresh solution was added before vortexing again for 5 min. Seeds were then rinsed several times with sterile water. To ensure this method effectively sterilized *D. ischaemum* seed, 100 seeds were placed on LB agar. No bacteria were observed after 10 days of incubation and a *Curvularia* sp. (determined through morphological characterization) was observed outgrowing from 2/100 seeds. 

Surface-sterilized, peeled, *D. ischaemum* seeds were placed on agarose and inoculated with bacteria in the same manner as described in Experiment 1. *T. officinale* seeds were prepared using the same methods as in Experiment 1 included in the experiment as a standard of comparison to Experiment 1. This experiment was conducted once, but was repeated with Isolates 4 and 5 within Experiment 3.

### 2.6. Experiment 3: Effect of Bacterial Isolates Alone and in Combination with Curvularia sp. on D. ischaemum, T. repens and P. annua Seedlings

The objective of these experiments was to evaluate the effect of certain bacterial isolates alone and in combination with a *Curvularia* sp. on germination and mortality of *D. ischaemum, P. annua* and *T. repens.* This experiment was conducted twice on separate dates. *P. annua* selected as it is a C_3_ grass and also as a model plant that can be used evaluate the effect of these treatments on root gravitropism and root length based on previous research [20,37].

Surface-sterilized *D. ischaemum, T. repens*, and *P. annua* seeds were placed on agarose and inoculated as described previously. Peeled *D. ischaemum* seeds were used in this experiment. Seeds were inoculated with bacterial isolates 4 and 5, the combination of 4 + 5, RiLB4 (standard), or no bacteria alone and in combination with a *Curvularia* sp. isolate collected outgrowing from *D. ischaemum* seed forming a factorial treatment design. Bacterial isolates were prepared and inoculated onto seeds in the same manner as described for Experiment 1; isolates 4 and 5 were combined by taking an aliquot of each bacterial suspension and combining them in a 1:1 ratio. A suspension of *Curvularia* conidia was prepared by gently washing a potato dextrose agar lawn culture with a solution of sterile water and 0.05% polysorbate 20 and lightly scraping with an inoculation loop. This resulted in a suspension that contained some hyphae but was primarily conidia. Four 10 µL aliquots were sampled and evaluated with a hemocytometer grid to determine the conidia concentration. Within 2 h of spore suspension preparation, a 2 or 4 µL drop (run A and B, respectively) was applied to each seed to inoculate 10^3^ ± 200 conidia per seed. The effect of each treatment on germination and seedling mortality was evaluated as previously described in Experiment 1. 

For each *P. annua* seedling, it was determined whether the root penetrated vertically into the agarose or grew horizontally along the surface of the agarose at 14 days after inoculation. Roots penetrating the agarose were determined to have a positive gravitropic response and the percentage of roots demonstrating a positive gravitropic response is presented [38]. *P. annua* root length was measured by removing the plant from the agarose and measuring the length of the primary root with a ruler.

### 2.7. Statistical Analysis

Experimental units were arranged in a completely randomized design in all experiments. For Experiments 1 and 2, a single-factor ANOVA was conducted using the GLM procedure in SAS (SAS Institute, Cary, NC, USA) v9.4 (α = 0.05). For Experiment 3, treatments were subjected to a factorial analysis with bacteria isolate and *Curvularia* inoculation serving as main effects. Means were separated using Fisher’s Protected LSD test at the 0.05 level. 

## 3. Results

### 3.1. Bacterial and Fungal Isolates

All isolates identified to the genus level had 16S rRNA sequences with >99% similarity to known sequences except for two (isolates 22 and 23) that had 94 and 95% similarity (Table 1). *Pantoea*, *Staphylococcus* and *Paenibacillus* spp. were isolated from non-surface sterilized *P. annua* seed. No bacteria or fungi were observed outgrowing from surface sterilized *P. annua* seed. A *Xanthomonas* and *Methylobacterium* sp. along with eleven *Pantoea* spp. were selected from surface sterilized *D. ischaemum* seeds. Two *Pantoea* isolates of interest (4 and 5) were identified as *Pantoea ananatis* based on both 16S sequence similarity to *Pantoea* spp. and *tuf* gene sequences with >99% similarity to *Pantoea ananatis*. Two unique fungal species were isolated and determined to be *Epicoccum* and *Curvularia* spp. through morphological characterization [37]. A *Curvularia* sp. was observed on 55% and 28% of the seeds in the first and second experimental run, respectively, and was isolated for further experimentation given its prevalence on surface-sterilized seed in this experiment and previous reports of *Curvularia* sp. affecting thermotolerance [24,25,26]. An *Epicoccum* sp. was observed outgrowing from 4 of 40 seeds in the first run and 10 out of 32 seeds in the second run.

### 3.2. Experiment 1: Effect of Bacterial Isolates on Taraxacum Officinale Seedling Mortality 

None of the bacteria isolated from *P. annua* seed caused *T. officinale* seedling mortality (Table 1). A *Staphylococcus* sp. isolate from *P. annua* caused minor *T. officinale* seedling injury. This experiment indicates that bacteria isolated from non-surface-sterilized *P. annua* seed are not pathogenic to *T. officinale* seedlings. 

While bacteria isolated from *P. annua* had limited or no effects on *T. officinale*, the most notable isolates from *D. ischaemum* (4, 5, 21, and 22) were *Pantoea* spp. that caused seedling mortality. Other isolates from *D. ischaemum* (8, 9, 10, 12, 21, 22, and 24) were not as antagonistic to *T. officinale* but still caused seedling injury and these were also *Pantoea* spp. while one isolate (18) was a *Xanthomonas* sp. These *Pantoea* spp. and *Xanthomonas* sp. were selected for futher study.

When these isolates were evaluated in a second experiment, isolates 5 and 8 caused the greatest *T. officinale* seedling mortality (Table 2). These isolates also caused injury to *T. repens* seedlings at 7 days after inoculation, and these seedlings likely would have completely died if observed 14 days after inoculation, as mortality of inoculated seedlings increased between 7 and 14 days after inoculation in subsequent experiments (reported herein). In previous research evaluating bacteria isolated from *Phragmites australis*, [20] demonstrated that *Pseudomonas fluorescens* strain Sandy LB4 caused >70% *T. officinale* seedling mortality while a *Pantoea* sp. strain RiLB4 did not increase mortality compared to the axenic control. In our experiments, Sandy LB4 nor RiLB4 increased *T. officinale* mortality compared to the bacteria free control.

**Figure 1:**
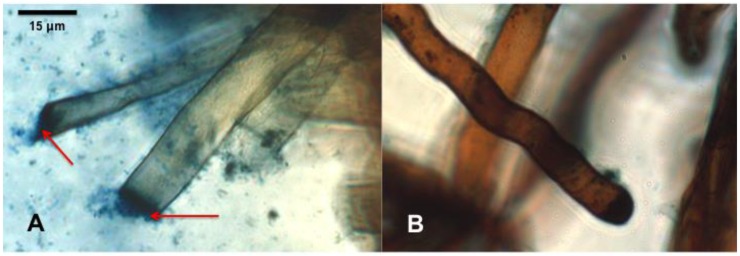
*Taraxacum officinale* seedling root hairs stained with aniline blue and DAB to visualize reactive oxygen species (H_2_O_2_ 14 days after seeds were inoculated with a *Pantoea* sp. (Isolate 4). Root hairs of an inoculated seedling (**A**) have ruptured while root hairs from a non-inoculated seed (**B**) are intact (indicated with arrows).

Bacteria were observed in squash preparations of *T. officinale* root tips treated with isolates 4 and 5. Root hairs of seedlings treated with isolates 4 and 5 were malformed and the membrane at the tip of the root hair was often completely destroyed where bacteria were present (Figure 1). Intact root hairs were observed in the axenic controls. Based on the results of this experiment, we selected isolates 4, 5, 8, 18, and 22 for further study.

### 3.3. Experiment 2: Effect of Bacterial Isolates on D. ischaemum Germination and Seedling Mortality

Isolate 22 and the combination of isolates 4 and 5 reduced *D. ischaemum* germination from 57% in the control to 33% and 20%, respectively (Table 3). Other isolates did not reduce germination compared to the bacteria free control. Isolates 8 and 22 increased *D. ischaemum* seedling mortality at 14 days after inoculation compared to the bacteria free control. Low germination prevented a proper assessment of the effect of the isolate 4 + 5 combination on *D. ischaemum* seedling mortality; these data were removed from the statistical analysis and are not presented. *T. officinale* was included to aid comparison to other experiments. All isolates except isolate 18 increased *T. officinale* mortality; a similar response was observed in Experiment 1. This indicates these bacteria are more pathogenic against competitor forbs than *D. ischaemum*. Based on the results of Experiments 1 and 2, we selected isolates 4 and 5 for further study.

### 3.4. Experiment 3: Effect of Bacterial Isolates alone and in Combination with Curvularia sp. on D. ischaemum, T. repens and P. annua Seedling Mortality

The effect of experimental run was not significant; therefore, the data from two experimental runs were combined. 

Germination of non-inoculated *P. annua* and *T. repens* was 93% and 83%, respectively and was not affected by bacteria or *Curvularia* treatment (data not presented). *D. ischaemum* germination was significantly reduced from 71% to 34% by *Curvularia*, but was not affected by bacterial treatment (data not presented). 

**Figure 2:**
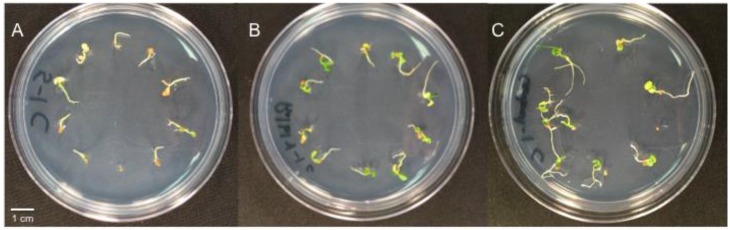
*Trifolium repens* seedlings 14 days after inoculation with *Pantoea* sp. Isolate 5 (**A**), *Pantoea* sp. Isolate RiLB4 (positive control) (**B**), and bacteria free control (**C**). Cotyledons inoculated with isolate 5 are pale and necrotic compared to the positive and bacteria free controls.

Bacteria and *Curvularia* treatment affected *T. repens* seedling mortality 2 weeks after inoculation (Table 4). Bacterial isolates 4 and 5 alone or in combination with each other caused between 57 and 81% *T. repens* mortality compared to 0 and 4% for RiLB4 and the bacteria free control (Figure 2). When inoculated with *Curvularia*, seedling mortality was similar across all treatments (64 to 87%). *Curvularia* also increased *D. ischaemum* mortality regardless of bacteria treatment. When averaged across bacterial treatments, *Curvularia* increased *D. ischaemum* mortality from 11% to 73%. In the absence of *Curvularia*, according to the statistical analysis, isolates 4 and 5 alone or in combination did not affect *D. ischaemum* mortality compared to RiLB4 and the bacteria free control. However, a non-significant trend suggests isolates 4 and 5 inoculated alone increase *D. ischaemum* seedling mortality, but seedlings are not affected by the mixture of isolates 4 and 5. The bacteria treatments did not affect *P. annua* mortality (data not presented). 

*Curvularia* increased *P. annua* mortality from 0 to 6% when averaged across all bacterial treatments, although this difference may not be biologically significant. *Curvularia* sp. treatment increased the positive gravitropic response of *P. annua* roots. When averaged across bacterial treatments, 57% of roots inoculated with *Curvularia* demonstrated a positive gravitropic response compared to 21% of roots without *Curvularia*. Bacteria treatments did not affect root gravitropism (data not presented). *Curvularia* nor bacteria treatments affected *P. annua* root length (data not presented).

### 3.5. Observation of Bacteria in Seedling Roots

*T. repens* and *T. officinale* root hairs stained with DAB after inoculation with isolate 5 were often malformed and bacteria were observed around root hairs but not usually in the cytoplasm; observations of root hairs treated with isolate 4 were similar except that L-forms of bacteria were occasionally observed in root hair cytoplasm (Figure 1). In every observation (*n* > 20) of DAB stained roots of seeds inoculated with bacteria, large numbers of bacterial rods were present in the rhizosphere, while no bacteria were observed in the rhizosphere of axenic controls. Conclusions from microscopic observations are limited, and the mechanism by which these bacteria kill *T. repens* and *T. officinale* should be investigated in more detail. 

## 4. Discussion

Our experiments demonstrate that *D. ischaemum* seed contains cultivable bacteria of the *Pantoea* genus and at least two cultivable fungi in genera *Epicoccum* and *Curvularia*. Certain *Pantoea ananatis* isolates caused more seedling mortality of competitor forbs *T. repens* and *T. officinale* than of *D. ischaemum* in axenic culture, indicating these isolates selectively antagonize forbs. This suggests that *D. ischaemum* seed harbors microbes that may antagonize competitor forbs. However, a natural environment in consortia with other microbes would likely affect the antagonistic characteristic of these microbes.

*Pantoea* spp. have been isolated as epiphytes from the phyllosphere of other plant species including *Oryza sativa* and *Phragmites australis* and can function as growth promoters, plant pathogens, and disease suppressors [20,39,40]. *P. ananatis* is often studied as a plant pathogen, but endophyte isolates can function as growth promoters in pepper and switchgrass (*Panicum virgatum* L.) [41,42,43,44]. Non-pathogenic *P. ananatis* endophyte isolates have also been found in sugarcane and *Miscanthus* x *giganteus* [45,46]. Most of these *P. ananatis* endophytes were found in root and shoot tissue. However, strains have been isolated from seedlings of surface-sterilized maize kernals [28]. Others investigated the function of three *P. ananatis* endophytes of maize seed and found one isolate was weakly pathogenic and other growth-promotional [29]. Despite several investigations into growth promotion and pathogenicity of host plants, we are not aware of any research demonstrating that *P. ananatis* can kill competitor plants. Research evaluating grass seed-associated bacteria as antagonists of competitor forbs is also limited. Others found that mixtures of certain bacteria associated with *Phragmites australis* seed increased mortality of *T. officinale* seedlings in axenic culture to a similar degree as bacteria evaluated in these experiments [20]. Previous research investigating deleterious rhizobacteria (for commercialization) evaluated bacterial strains further if they reduced root length of the target and non-target species by more than 30% and 10%, respectively, in Petri dish culture [47,48,49]. We did not measure forb root length in our experiments, but forb: *D. ischaemum* mortality ratios ranged from 2:1 to 5:1.

This research demonstrates that *Pantoea* spp. isolated from *D. ischaemum* seed have the capacity to antagonize seedlings of competitor forbs in axenic culture. *Pantoea ananatis* isolates consistently caused necrosis indicative of membrane leakage in *T. repens* and *T. officinale* cotyledons beginning about 7 days after inoculation and progressing until complete death was observed 14 to 21 days after inoculation. In our research, RiLB4 was included as a standard and did not increase mortality of *T. repens* and *T. officinale* while several *Pantoea* spp. isolates from *D. ischaemum* caused 50 to 80% mortality of *T. repens* and *T. officinale* seedlings. The effect of these isolates on *D. ischaemum* seedlings was inconsistent, but results indicate that they may increase seedling mortality. However, in some experiments, mortality was reduced when isolates 4 and 5 were combined, thus, this combination demonstrated the greatest amount of selectivity for the competitor forbs. It is also possible that removing the *D. ischaemum* seed coat (required to surface sterilize the seed) increased *D. ischaemum* susceptibility to these bacteria and reduced selectivity for the competitor forbs. Future research should evaluate whether these bacteria are less pathogenic when in consortium with one another or other uncultivable bacteria.

**Figure 3:**
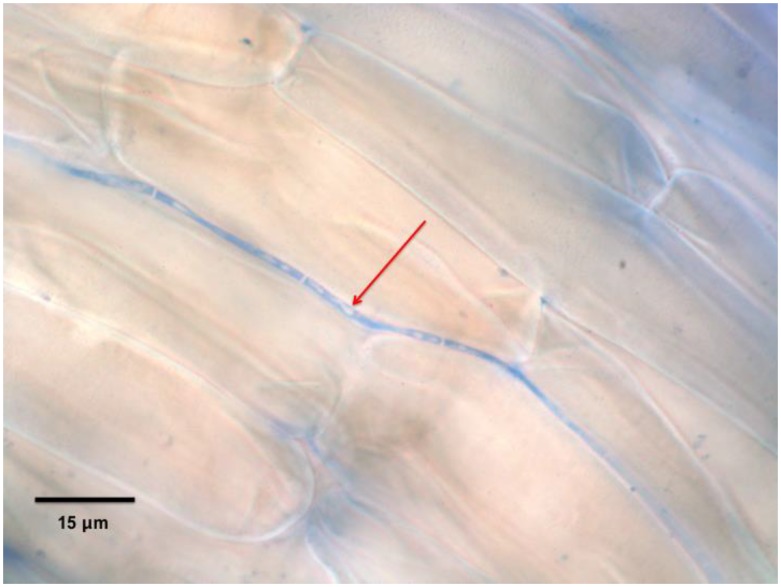
Root cortex cells in the primary *Poa annua* seedling root 10 days after seeds were inoculated with a *Curvularia* sp. showing an intercellular fungal hypha (indicated with arrows) stained with aniline blue (0.1%).

In our experiments, *Curvularia* functioned primarily as a pathogen of *T. repens* and *D. ischaemum*, although with a different combination of bacterial cohorts it may function as a symbiont [10]. *Curvularia* was much less pathogenic to *P. annua* than *D. ischaemum* and increased the positive gravitropic response of *P. annua* roots. To potentially avert the pathogenic effect against *D. ischaemum*, future research could use more mature seedlings, perhaps allowing plants to develop at least one true leaf before inoculating with this *Curvularia* sp. isolate to evaluate stress responses similar to [25] and [26]. Extensive observations of seeds inoculated with *Curvularia* sp. were not conducted in these experiments, but in every observation on multiple dates, segmented hyphae was observed colonizing intercellular spaces of inoculated *P. annua* and *D. ischaemum* root cortex tissue, which suggests it has endophytic capabilities (Figure 3).

These experiments demonstrate that *Digitaria ischaemum* seeds contain bacteria that antagonize competitor species in Petri dish experiments. *Pantoea ananatis* may provide *D. ischaemum* a competitive advantage against competitor species. The effect of these isolates alone and in consortia on *D. ischaemum* as compared to competitor forbs should be investigated further. Future research could inoculate these bacteria onto *D. ischaemum* seeds to determine they affect competitiveness.

One limitation of this research is that seeds were collected from only one location for each plant species. Plant microbes likely vary across geographic sites and genotypes. Future research could investigate bacteria associated with *P. annua* and *D. ischaemum* seeds collected from different geographic and climatic regions using culture-dependent and culture-independent methods.

The mechanism of selective pathogenicity should also be elucidated. Cyanide production has been implicated as a mechanism by which rhizobacteria suppress plant growth [50], but preliminary experiments suggest that our *Pantoea* isolates do not produce cyanide. Interestingly, we consistently observed cotyledons with a water-soaked appearance prior to necrosis and death. Similar symptoms can be observed in corn plants affected by *Pantoea stewartii* subsp. *stewartii*, (the causal agent of Stewart’s wilt) as a result of biofilms encased in an exopolycaccharide [51]. However, we did not observe exopolycaccharide blockages in the xylem tissue. The role of Large *Pantoea* Plasmids in the phenotype and selective forb control we observed should be investigated. These plasmids are common to many *Pantoea* spp. including *P. ananatis* are thought to be responsible for certain strains to occupy specific ecological niches with various phenotypes such as toxin production and viruluence factors that enable pathogenicity or biocontrol [52,53,54,55]. The proteins encoded by Large *Pantoea* Plasmids may shed light on the bacterial mechanism of action. 

## Figures and Tables

**Table 1:** Effect of bacteria collected outgrowing from surface-sterilized *Digitaria ischaemum* seeds and non-surface-sterilize *Poa annua* on *Taraxacum officinale* seedling mortality in axenic agarose culture 14 days after inoculation. Isolates 1 to 6, 7 to 12, 13 to 18, and 19 to 24 were evaluated in separate experiments in Run A. A non-treated control was included in each experiment. Sandy LB4 and RiLB4 (Genbank No. KX752781 and KX665565, respectively) were included as standards and evaluated with isolates 7 to 12.

Isolate No.	Isolated from	Seedling Mortality (%) ^†^	Seedlings Injured (%)
1	*P. annua*	0	21
2	*P. annua*	0	5
3	*P. annua*	0	7
4	*D. ischaemum*	19 *^,§^	44 *
5	*D. ischaemum*	47 *	87 *
6	*P. annua*	0	6
No bacteria	*-*	0	18
7	*P. annua*	0	11
8	*D. ischaemum*	29	69 *
9	*D. ischaemum*	13	42 *
10	*D. ischaemum*	8	67 *
11	*P. annua*	0	30 *
12	*D. ischaemum*	10	67 *
No bacteria	*-*	0	3
13	*D. ischaemum*	21	54
14	*P. annua*	0	31
15	*P. annua*	0	16
16	*D. ischaemum*	10	27
17	*D. ischaemum*	14	52
18	*D. ischaemum*	23	40
No bacteria	*-*	0	22
19	*P. annua*	0	45
20	*D. ischaemum*	0	41
21	*D. ischaemum*	39 *	80 *
22	*D. ischaemum*	37 *	79 *
23	*D. ischaemum*	0	36
24	*D. ischaemum*	0	46 *
RiLB4	*P. australis*	0	11
Sandy LB4	*P. australis*	8	35
No bacteria	-	0	38

^†^ Percent mortality is the percentage of emerged seedlings that were determined by visual assessment to be completely dead. Seedlings were scored as injured if they displayed some injury symptoms but were not completely dead. ^§^ An asterisk indicates that the level of mortality or injury was significantly different from the non-treated control according to Fisher’s Protected LSD test (α = 0.05). Isolates were evaluated in four separate experiments.

**Table 2:** Effect of bacteria collected outgrowing from surface-sterilized *Digitaria ischaemum* seeds on *Taraxacum officinale* seedling mortality 14 days after inoculation and *Trifolium repens* 7 days after inoculation in axenic agarose culture. Only certain isolates were evaluated based on the results of Run A. Isolates RiLB4 and Sandy LB4 (Genbank No. KX752781 and KX665565, respectively) were isolated from *Phragmites australis* and were included as standards.

	*T. officinale*		*T. repens*	
Isolate No.	Seedling Mortality (%) ^†^	Seedlings Injured (%)	Seedling Mortality (%)	Seedlings Injured (%)
4	12	49 *	16 *	88 *
5	39 *^,§^	68 *	5	76 *
8	17 *	42 *	8	83 *
10	3	19	8	84 *
12	11	33 *	12	93 *
13	11	22	8	78 *
17	4	40 *	7	67 *
18	0	29 *	49 *	84 *
21	4	17	7	89 *
22	4	40 *	0	96 *
RiLB4	-	-	0	36
Sandy LB4	0	22	0	23
None	0	4	0	34

^†^ Percent mortality is the percentage of emerged seedlings that were determined by visual assessment to be completely dead. Seedlings were scored as injured if they displayed some injury symptoms but were not completely dead. ^§^ An asterisk indicates that this level of mortality was significantly different from the non-treated control according to Fisher’s Protected LSD test (α = 0.05).

**Table 3:** Effect of *Pantoea* spp. isolates on *Digitaria ischaemum* germination and seedling mortality 14 and 28 days after inoculation in axenic agarose culture. Ten seeds were placed in each Petri dish and each treatment was replicated in three Petri dishes. *Taraxacum officinale* seeds were also inoculated in separate Petri dishes and included for comparison; mortality was evaluated at 21 days after treatment.

Bacterial Isolate	Germination (%)		Seedling Mortality (%) ^†^				
	*D. ischaemum*		*D. ischaemum*			*T. officinale*	
	14 days		14 days		28 days	21 days	
4	40	abc ^§^	0	b	29	71	a
5	53	ab	6	ab	13	86	a
8	43	ab	18	a	43	78	a
18	43	ab	0	ab	7	33	b
22	33	bc	19	a	25	79	a
4 + 5	20	c	-	-	-	72	a
none	57	a	0	b	0	17	b

^†^ Percent mortality is the percentage of emerged seedlings that were determined by visual assessment to be completely dead. ^§^ Means followed by the same letter are not significantly different according to Fisher’s Protected LSD test (α = 0.05). In columns where no letters are present, the ANOVA determined that the treatment effect was not significant (α = 0.05).

**Table 4:** Effect of *Pantoea ananatis* isolates 4 and 5 alone and in combination with *Curvularia* sp. spores outgrowing from surface-sterilized *Digitaria ischaemum* seeds on *Trifolium repens* seedling mortality 14 days after inoculation in axenic agarose culture. Ten seeds were placed in each Petri dish and each treatment was replicated in three Petri dishes per experimental run. Combined results of two experimental runs are presented. A *Pantoea* sp. (RiLB4; Genbank No. KX752781) isolated from *Phragmites australis* and was included as a standard.

Bacterial Isolate	*Curvularia*	Seedling Mortality (%) ^†^			
		*T. repens*		*D. ischaemum*	
4	no	67	a ^§^	19	b
5	no	81	a	31	b
4 + 5	no	57	a	2	b
RiLB4	no	0	b	0	b
none	no	4	b	3	b
4	yes	73	a	77	a
5	yes	87	a	63	a
4 + 5	yes	87	a	68	a
RiLB4	yes	64	a	77	a
none	yes	73	a	83	a

^†^ Percent mortality is the percentage of emerged seedlings that were determined by visual assessment to be completely dead. ^§^ Means followed by the same letter are not significantly different according to Fisher’s Protected LSD test (α = 0.05).
